# Noncanonical interactions between serpin and β‐amylase in barley grain improve β‐amylase activity *in vitro*

**DOI:** 10.1002/pld3.54

**Published:** 2018-05-08

**Authors:** Maja Cohen, Robert Fluhr

**Affiliations:** ^1^ Department of Plant Sciences Weizmann Institute of Science Rehovot Israel

**Keywords:** amylase, barley, enzyme stability, *Hordeum vulgare*, oxidative stress, protein interaction, serpin

## Abstract

Serpin protease inhibitors and β‐amylase starch hydrolases are very abundant seed proteins in the endosperm of grasses. β‐amylase is a crucial enzyme in the beer industry providing maltose for fermenting yeast. In animals and plants, inhibitory serpins form covalent linkages that inactivate their cognate proteases. Additionally, in animals, noninhibitory functions for serpins are observed such as metabolite carriers and chaperones. The function of serpins in seeds has yet to be unveiled. In developing endosperm, serpin Z4 and β‐amylase showed similar *in vivo* spatio‐temporal accumulation properties and colocalize in the cytosol of transformed tobacco leaves. A molecular interaction between recombinant proteins of serpin Z4 and β‐amylase was revealed by surface plasmon resonance and microscale thermophoresis yielding a dissociation constant of 10^−7^ M. Importantly, the addition of serpin Z4 significantly changes β‐amylase enzymatic properties by increasing its maximal catalytic velocity. The presence of serpin Z4 stabilizes β‐amylase activity during heat treatment without affecting its critical denaturing temperature. Oxidative stress, simulated by the addition of CuCl_2_, leads to the formation of high molecular weight polymers of β‐amylase similar to those detected *in vivo*. The polymers were cross‐linked through disulfide bonds, the formation of which was repressed when serpin Z4 was present. The results suggest an unprecedented function for a plant seed serpin as a β‐amylase‐specific chaperone‐like partner that could optimize β‐amylase activity upon germination. This report is the first to describe a noninhibitory function for a serpin in plants.

## 1 INTRODUCTION

Serpin proteins are part of a well‐conserved superfamily of proteins in plants and animals. All serpin types share a similar secondary structure, although their specific amino acids sequence can differ. Serpins are metastable proteins and undergo conformational changes, from “stressed” to “relaxed” transition to complete their various functions (Huntington, Read, & Carrell, 2000). They are mostly known as serine protease inhibitors in animal cells (Gettins, 2002) and as cysteine protease inhibitors controlling cell death in plant cells (Fluhr, Lampl, & Roberts, 2012; Lampl, Alkan, Davydov, & Fluhr, 2013; Lampl et al., 2010; Lema Asqui et al., 2017; Vercammen et al., 2006). The secondary structure of a serpin includes a reactive center loop (RCL) domain that acts as bait for its cognate protease by mimicking the preferred P1‐P1′ digestion site (Khan et al., 2011). Other functions for serpins were discovered in animal systems. The noninhibitory serpins do not exhibit protease inhibitory properties and instead have a panel of activities including: hormone transport by the serpin thyroxine‐binding globulin (Pemberton, Stein, Pepys, Potter, & Carrell, 1988); chaperone‐like functions by SERPINH1 binding to collagen (Sauk, Nikitakis, & Siavash, 2005) and as storage proteins for ovalbumin, in egg white (Stein et al., 1990). In contrast, the myeloid and erythroid nuclear termination (MENT) serpin retains protease inhibition but also functions in chromatin remodeling (Irving et al., 2002). In plants, noninhibitory functions for serpins have yet to be characterized.

Serpins are present in seeds of cereals such as barley, wheat, oat, and rye (Hejgaard, 2001; Hejgaard & Hauge, 2002; Østergaard, Finnie, Laugesen, Roepstorff, & Svennson, 2004; Rasmussen, Dahl, Nørgård, & Hejgaard, 1996; Rosenkrands, Hejgaard, Rasmussen, & Bjørn, 1994). In barley grains, the two major types, serpin Z4 and serpin Z7, represent up to 5% of the total seed proteome (Evans & Hejgaard, 1999). The amounts of the two proteins vary among the various cultivars (Evans & Hejgaard, 1999; Finnie et al., 2004). In wheat and oat, the RCL sequence of the grain serpins contains glutamines, a very abundant amino acid in storage proteins (Ostergaard, Rasmussen, Roberts, & Hejgaard, 2000). Therefore, it was suggested that serpins could protect grain storage proteins by inhibiting predator's proteases adapted to the proteolysis of glutamine‐rich substrates. However in barley, the RCL sequence of serpin Z4 and Z7 is not similar to any of the main storage protein sequences, and, although both serpins are protease inhibitors of cathepsin G and trypsin, their inhibitory activity appears to be less potent than other plant serpins (Dahl, Rasmussen, & Hejgaard, 1996). Alternatively, considering their abundance in the endosperm, serpins were suggested to have a role as storage proteins. However, serpins in barley grains are relatively resistant to degradation by proteases during germination. Indeed, the level of the soluble serpin continues to increase 2 days after seed imbibition (Bønsager, Finnie, Roepstorff, & Svensson, 2007). Hence, the function of barley grains serpins remains an open question.

In seeds, serpins exist as two distinct forms, the free serpins, extractable with water‐based buffer and the bound fraction of serpins, extractable only with thiol enriched buffers (Evans & Hejgaard, 1999). β‐amylase, another very abundant endosperm protein, also exists in these two forms (Evans, Wallace, Lance, & MacLeod, 1997; Evans, MacLeod, et al., 1997). The ratio between bound and free β‐amylase can be modulated by the presence of a cysteine at position 115 (Li et al., 2002). This position is also critical for conferring distinct IEF band patterns (Ma, Langridge, Logue, & Evans, 2002) and protein thermostability (Ma, Evans, Logue, & Langridge, 2001; Ma, Stewart, et al., 2000). β‐amylase accumulates as a water‐insoluble protein during grain ripening but during germination or malting becomes readily extractable (Finnie, Melchior, Roepstorff, & Svensson, 2002; Finnie et al., 2004). It is thought that cysteines play a key role in these changes. For example during germination, the C‐terminal region of β‐amylase that contains a cysteine is removed by limited proteolysis (Guerin, Lance, & Wallace, 1992; Lundgard & Svensson, 1986). In addition, two cysteines are targeted and reduced by thioredoxins (Hagglund et al., 2010). The combined action of proteases and reducing agent released by the aleurone layer convert the bound form of β‐amylase in the endosperm to its free form. Serpin and β‐amylase are both found in the endosperm tissue (Finnie & Svensson, 2003; Roberts, Marttila, Rasmussen, & Hejgaard, 2003) adhering to the border of starch granules (Borén, Larsson, Falk, & Jansson, 2004; Hara‐Nishimura, Nishimura, & Daussant, 1986; Wang et al., 2011). Upon gel filtration of salt extract from barley dry grain, serpin and β‐amylase eluted in high molecular weight fractions prompting speculation of their tight association (Hejgaard & Carlsen, 1977).

Although the exact function of β‐amylase in natural seed physiology is not known, it is a critical enzyme in the beer industry; it produces highly fermentable maltose disaccharides through the hydrolysis of the α‐1,4‐glycosidic bond of polysaccharide chains (MacGregor, Bazin, Macri, & Babb, 1999). Moreover, as one of the most abundant barley protein and as one of the only residual beer proteins, serpin has been investigated for its impact on beer quality (Iimure, Nankaku, Kihara, Yamada, & Sato, 2012; Iimure & Sato, 2013; Specker, Niessen, & Vogel, 2014). In the results presented here, we show that serpin and β‐amylase interact in a quantifiable manner. The interaction enhances β‐amylase enzymatic properties and increases β‐amylase stability to heat and oxidative stress. This research suggests an unprecedented noncanonical function for serpin in plants as a β‐amylase‐specific molecular chaperone.

## 2 MATERIALS AND METHODS

### 2.1 Plant material

Barley cv. Harrington was grown in the greenhouse under 24/20°C (day/night) at natural day length conditions. For sampling of developing grain, flowering kernels were marked and sampled at the indicated times. For germination experiments, barley grains were imbibed in water for 24 h and placed on wet filter paper at 22°C under natural day length conditions. The grains were sampled at the indicated time without the coleoptile and the radicle.

### 2.2 Gel fractionation and immunoblots

Grains at different growth stages were ground in liquid nitrogen. Tissue (100 mg) was dissolved in 1 ml of Dulbecco's phosphate‐buffered saline (DPBS without calcium and magnesium) and plant protease inhibitor cocktail (Sigma P9599) with or without 20 mM DTT. The extracted tissue was left shaking for 15 min at room temperature and centrifuged for 15 min at 17,000 ***g*** at 4°C. Gel fractionation on denaturing or nondenaturing gels was as described (Lampl et al., 2010). The membrane was developed with β‐amylase antibodies (1:1000) and secondary anti‐rabbit horseradish peroxidase (1:3000) or with serpin Z4 antibodies (1:1000), and secondary anti‐rat horseradish peroxidase (1:3000). For purified proteins, the proteins were first dialyzed in PBS and then mixed with reducing or oxidizing buffers, left at room temperature for 10 min, and then fractionated onto 10% SDS‐PAGE. Purified serpin Z4 was used to generate antibodies from rat serum. Antibodies against β‐amylase were purchased from antibody‐online (cat # ABIN285322).

### 2.3 Cloning and transient expression in *Nicotiana benthaniama*

Serpin Z4 and β‐amylase were cloned from cDNA of barley cv. Harrington. The serpin and the amylase were fused to an RFP on the N terminus and a GFP on the C‐terminus, respectively, under a 35S promoter in vector pART27 and introduced into the plant transformation vector pML*‐*BART (Pogorelko, Fursova, Ogarkova, & Tarasov, 2007). The primers used for the cloning are shown in Table S1. The plasmid was expressed in Agrobacterium GV301 and infiltrated into 1‐month‐old leaves of *N. benthamiana*. All images were taken with a model no. A1 Confocal Microscope (Nikon, Melville, NY) GFP emission/excitation 525/488 and RFP emission/excitation 595/561.

### 2.4 Protein expression and purification

A construct encoding β‐amylase (WT and mutants) or serpin Z4 isolated from barley cv. Harrington was cloned into the pET28‐(a) vector (Novagen) with a C‐terminal hexahistidine tag. The primers used for the cloning are shown in Table S1. Expression was induced in *Escherichia coli* SHuffle cells (Lobstein et al., 2012) grown to an OD_600_ of 0.6–0.7 at 28°C then supplied with 1 mM isopropyl‐β‐d‐thiogalactoside and cultured for 16 h at 16°C. Cells were resuspended in lysis buffer (0.2 M NaH_2_PO_4_, 500 mM NaCl, 10 mM imidazole, 1 mM DTT pH 7.4) and lysed by sonication. Cleared lysate was purified by Ni‐NTA affinity chromatography. The eluate was concentrated and purified further by gel filtration (Superdex 200 Increase 10/300GL; GE Healthcare) in PBS buffer supplied with 2 mM DTT. The purified proteins were aliquoted and stored at −80 in 5% glycerol. Wild‐type β‐amylase was analyzed offline on a Q‐Trap 4000 (Sciex) operated in the positive ion mode. All samples were diluted into 50% methanol/5% formic acid and loaded into Nano spray borosilicate emitters. Typical instrument settings for Q1 MS were applied.

### 2.5 Chymotrypsin inhibition assay

Peptidase activity of 70 ng of chymotrypsin (Sigma C4129) was tested on casein substrate with serpin BsZx or Z4 at a molar ratio of 2:1, serpin: protease. Proteolytic activity was measured using Pierce™ Fluorescent Protease Assay Kit (cat# 23266).

### 2.6 Biacore and Microscale thermophoresis assays

Purified proteins (serpin Z4 and β‐amylase) were first immobilized via amine coupling to the free carboxyl groups on the CM5 chip surface following standard BIAcore protocols. Next, the reciprocal protein (either serpin Z4 or β‐amylase) was injected at various concentrations to generate kinetics sensorgrams. The experiments were carried out in PBS buffer on a Biacore T200 instrument (GE Healthcare Life Sciences).

Microscale thermophoresis analysis was performed on a NanoTemper Monolith NT.115 instrument (NanoTemper Technologies). β‐amylase and serpin Z4 were fluorescently labeled with a NHS‐Ester Fluorescent Dye. Labeled β‐amylase (70 nM) was mixed with varying concentration of unlabeled serpin Z4 (from 40 μM to 1 nM) and 70 nM of labeled serpin Z4 with 16 μM to 0.5 nM of unlabeled β‐amylase. Each solution was transferred into a standard glass capillary soon after mixing and immediately analyzed using settings of 20% power and 50% excitation power. For each set of binding experiments, three independent MST measurements were carried out. Data analysis was performed by the Nanotemper analysis software.

### 2.7 β‐amylase activity assays

Purified β‐amylase was first dialyzed in PBS to remove DTT using centrifugal filter (Amicon ultra, millipore). β‐amylase (2 or 2.5 pmol) in 10 μl PBS was mixed with 20 μl of 2% starch in 25 mM NaAc pH 4.8 and incubated for 10 min at 25°C. The reaction was terminated by the addition of 30 μl of 3,5‐dinitrosalicylic acid (DNS) solution (Miller, 1959) then boiled for 5 min. After cooling, 250 μl of distilled water was added, and the absorbance was measured at 540 nm (microplate reader TECAN Infinite 200) with maltose as the standard. Amylase activity is expressed in nmol maltose per minutes. For additives, 2 or 2.5 pmol of β‐amylase in 5 μl was mixed with 5 μl of 2× solution containing either serpin Z4, DTT, or CuCl_2_ and left at room temperature for 10 min. Results were analyzed by linear regression performed by GraphPad Prism software. The specific measurement of β‐amylase activity in grains was measured using a megazyme^®^ β‐amylase assay kit (Betamyl‐3; Megazyme, Australia) following the manufacturer's instructions.

### 2.8 Thermal shift assay

β‐amylase (5–0.5 μM) in 2.5 μl was mixed with 2.5 μl of a 1:5 molar ratio of serpin Z4. PBS buffer (10 μl) and 5 μl of 4× sypro‐orange solution (S5692; Sigma) were added to the protein and inserted into a real‐time polymerase chain reaction device (StepOne™ Real‐time PCR System) using 96‐well plate in four replicates. The settings were as follow: Starting temperature was 25°C, and the fluorescence data were collected every 2°C until 99°C. The results were analyzed using protein thermal shift software provided by Thermo Fisher Scientific.

## 3 RESULTS

### 3.1 Serpin and β‐amylase coexpress during development and colocalize to the cytoplasm

**Figure 1:**
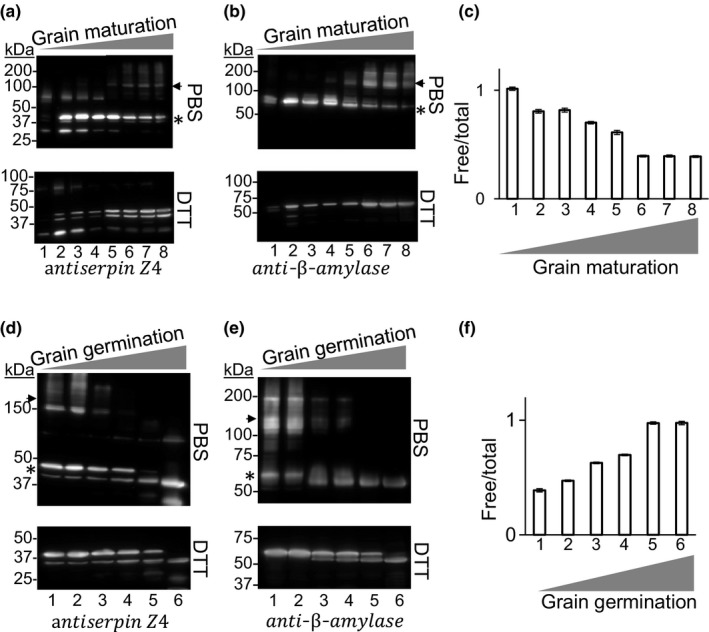
Accumulation of β‐amylase and serpin Z4 during grain maturation and germination. Each Western blot was replicated at least three times using independent biological replicates. (a–c) Proteins were extracted from developing grains of barley cv. Harington with either PBS (upper blots) or PBS with 20 mM DTT (lower blots). In a and b, the proteins were fractionated on a nonreducing SDS‐PAGE gel transferred to a PVDF membrane and blotted against serpin Z4 in a or against β‐amylase in b. Lanes are days after pollination (DAP) Lanes 1–8; 8, 13, 18, 22, 28, 32, 39, dry grains. (c) β‐amylase activity was assayed on the same samples using the Betamyl‐3 method, shown is the ratio of free β‐amylase activity over total β‐amylase activity. (d–f) Proteins were extracted from 100 mg of germinating grains of barley cv. Harington with 1 ml of either PBS (upper blots) or PBS with 20 mM DTT (lower blots). Lanes are hour after imbibition (HAI) from left to right 1–6; 0, 5, 24, 30, 48, and 120 HAI. (d and e) Proteins were loaded on a nonreducing SDS‐PAGE gel transferred to a PVDF membrane and blotted against serpin Z4 in d and against β‐amylase in e. f, β‐amylase activity was assayed on the same grain samples as carried out in (c). The sizes of the proteins are indicated in kDaltons (kDa). The asterisk indicates the monomer size of serpin and β‐amylase, and the arrow indicates high molecular weight aggregates of the proteins

Specific antibodies were used to concurrently analyze by immunoblot the accumulation of serpin and β‐amylase during grain development and germination in barley cv. Harrington. In developing grain, under fractionation in reducing conditions (1,4‐dithiothreitol, DTT), polypeptides of approximately 40 and 60 kDa for serpin Z4 and β‐amylase (Figure 1a and b lower blots) were detected, respectively. A gradual increase in both proteins was observed during the first 30 days followed by a plateau phase lasting until the grain was dry. In nonreducing conditions, in which pre‐existing disulfide bonds are maintained, the monomeric forms of both serpin Z4 and β‐amylase (Figure 1a and b upper blots) accumulated from 8 DAP to 28 DAP. They decreased from 28 DAP to the stage of “dry grains” while higher molecular weight forms of these polypeptides accumulated (100–200 kDa). Enzymatic activity of β‐amylase was determined in the free (water soluble) and total (thiol extractable) fraction of the developing grains and is represented by the ratio of free β‐amylase activity over total β‐amylase activity. A decrease in the free/total β‐amylase activity was recorded during grain development that parallels the accumulation of bound β‐amylase and the appearance of higher molecular weight complexes.

In a similar manner, the content of serpin and β‐amylase was investigated during germination. Under reducing conditions (Figure 1d and e; lower blots), the full‐length protein and a processed form are observed. The processed size of serpin Z4 corresponds to the size of a spent serpin that has interacted with an unknown protease at the RCL (Gettins, 2002). The size of the processed β‐amylase (55 kDa) coincides with the size of β‐amylase after partial proteolysis of the C‐terminal portion. Indeed, proteases released from the aleurone layer are known to target the β‐amylase C‐terminus (Guerin et al., 1992; Lundgard & Svensson, 1986). Fractionation of proteins without reducing agent show that the 200 kDa size migrating polypeptides disappeared between 5 and 24 HAI (Figure 1d and e; upper blots). The disappearance of high molecular weight proteins is consistent with reports of increasing accumulation of thioredoxin reductive activity (Hagglund et al., 2010; Lundgard & Svensson, 1986). In a reciprocal manner to what was observed in developing seeds, the ratio of free over total enzymatic activity of β‐amylase increased in parallel with the disappearance of higher molecular weight forms (Figure 1f).

**Figure 2:**
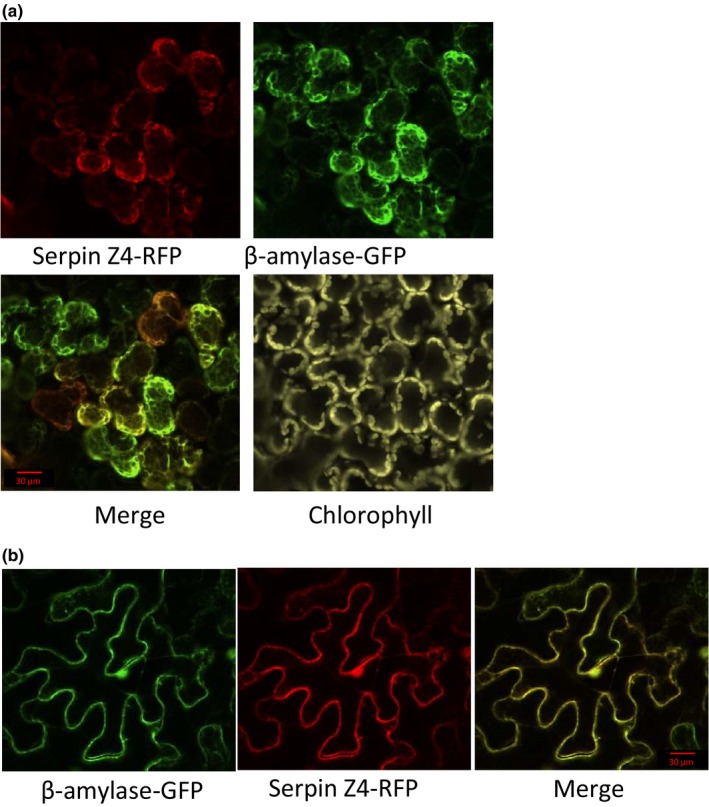
Transient expression of serpin Z4‐RFP and β‐amylase‐GFP in leaves of *Nicotiana benthaniama*. Confocal microscope observation of a, spongy mesophyll cells and b, epidermal cells

The results underscore correlation between the accumulation patterns of serpin Z4 and β‐amylase during seed development and germination. Therefore, it was of interest to examine their cellular localization as well. Plant serpins are known to reside either in the cytoplasm or are secreted to extracellular spaces (Fluhr et al., 2012), while β‐amylase can localize to the chloroplast (amyloplast) or cytoplasm in Arabidopsis leaves (Mita, Suzuki‐Fujii, & Nakamura, 1995; Sparla, Costa, Schiavo, Pupillo, & Trost, 2006). Agrobacterium harboring constructs with serpin Z4 fused to RFP and β‐amylase fused to GFP were coinfiltrated into *N. benthamiana* plants. To verify the reliability of this assay, immunoblot analysis showed that the expressed protein sizes were commiserated with their expected fused size (Fig. S1). As shown in Figure 2, the confocal images of both serpin Z4 and β‐amylase colocalized to the cytoplasm surrounding the chloroplasts of spongy mesophyll (Figure 2a). They are also expressed in the cytoplasmic peripheral regions of leaf epidermal cells (Figure 2b).

### 3.2 Serpin Z4 and ß‐amylase exhibit quantifiable protein–protein interactions

To examine for possible interaction between serpin Z4 and β‐amylase, their recombinant proteins were first purified by affinity chromatography and size exclusion chromatography. The gel image of the proteins is shown in Fig. S2. The proteins were further tested for conventional activity. The activity of serpin as protease inhibitor was established by calculating its inhibition factor, *X*_inh_. This factor measures the fraction of complex forming serpin in equilibrium with the protease as determined by the residual peptidase activity. As shown in Fig. S3a, recombinant serpin Z4 inhibits chymotrypsin with a *X*_inh_ of 0.35 while a different barley recombinant serpin, BsZx inhibited the protease with a ratio of 0.88. The results are consistent with that measured previously for isolated barley serpins (Dahl et al., 1996). Recombinant amylase activity was measured using two independent assays; the DNSA reducing sugar method (Miller, 1959) and the Betamyl‐3 method as described in the Material and Methods. Figure S3c and d shows that recombinant β‐amylase activity in both methods was linear from 1 to 8 pmol of enzyme, similar to what was reported previously (Lundgard & Svensson, 1987).

**Figure 3:**
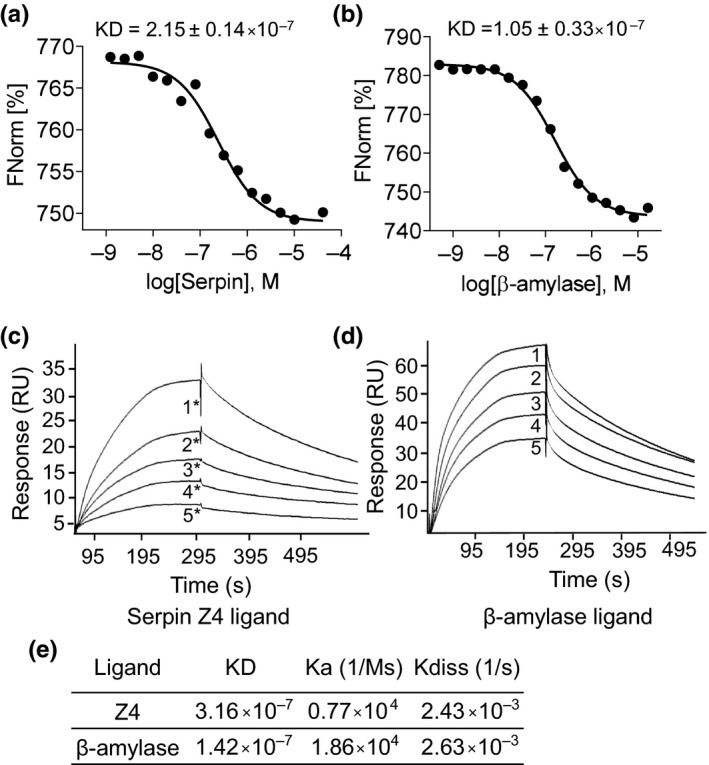
Interaction between serpin Z4 and β‐amylase established by microscale thermophoresis and by surface plasmon resonance. (a) β‐amylase was bound to a fluorescent probe, and increasing amounts of serpin Z4 were added. (b) Fluorescent serpin Z4 was probed with increasing β‐amylase as in a. The assays were conducted in PBS buffer with 0.1% Tween‐20. For estimation of KD, the results of three replicate experiments were combined. (c) Serpin Z4 was attached to a Biacore compatible sensor chip, and β‐amylase was injected at the following concentrations: 2 μM, 0.9 μM, 0.4 μM, 0.2 μM, and 0. 1 μM (1* to 5*, respectively). (d) β‐amylase was attached to the sensor chip and serpin Z4 injected at the following concentrations: 0.6 μM, 0.5 μM, 0.4 μM, 0.3 μM, and 0.2 μM (1–5, respectively). (e) Equilibrium constants of the interaction measured by surface plasmon resonance

Serpin Z4 and β‐amylase activities were reported to comigrate during gel filtration of barley seed extracts (Hejgaard & Carlsen, 1977). This could be a result of their interaction or stem from similar molecular weights due to self‐oligomerization. To examine this directly, we employed microscale thermophoresis as shown in Figure 3. Microscale thermophoresis measures the motion of molecules along a microscopic temperature gradient (Jerabek‐Willemsen, Wienken, Braun, Baaske, & Duhr, 2011). A fluorescent label was attached covalently, to one of the proteins to be tested and added to a serial dilution of the unlabeled cognate protein (X axis in log [M]; see Materials and Methods; Figure 3). The change in motion is presented as normalized fluorescence intensity (Y axis). Typical MST traces and capillary shapes are shown in Fig. S4. The KD of this interaction was found to be 2 · 10^−7 ^M or 1 · 10^−7 ^M depending on the direction of the reciprocal labeling (Figure 3a and b). The results quantify a physical interaction between serpin and β‐amylase in native nonreducing conditions with an affinity of approximately 10^−7 ^M.

The interaction of β‐amylase and serpin Z4 was further measured by surface plasmon resonance using a BIAcore sensor chip (see Materials and Methods; Figure 3c and d). When serpin was immobilized on the surface and β‐amylase was the analyte the recorded sensorgram yielded a binding affinity constant (KD) of 3.16 · 10^−7 ^M. Likewise, when the proteins were interchanged and β‐amylase was immobilized the affinity measured was 1.42 · 10^−7^ M. As shown in Figure 3e, the KD is the result of the ratio of the two rate constants, where Ka and Kdiss were ~10^4^ and 10^−3^, respectively. The relatively low Ka indicates a slow association rate and the relatively high Kdiss, a fast dissociation process. Thus, two diverse biophysical methods of measurement show that β‐amylase and serpin Z4 display a significant measurable interaction with a binding affinity constant greater than 10^−7^ M. Although the motivation for this experiment relied on the observation that serpin Z4 and β‐amylase comigrate during gel filtration, the consensus KD of 10^−7^ M measured here would be too low to form stable complexes during gel filtration. Hence, it is possible that previous observation of comigration emanated from the formation of homomultimers yielding similar filtration qualities (see below).

### 3.3 Serpin interaction with β‐amylase impacts on its enzymatic properties

**Figure 4:**
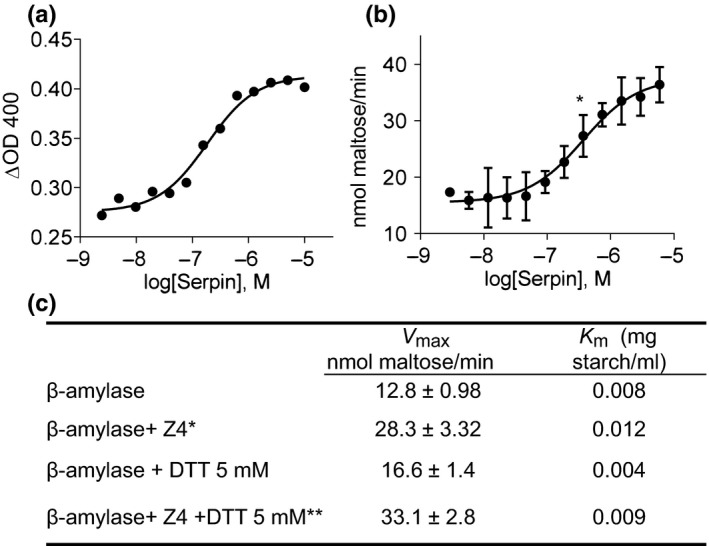
Activity of β‐amylase in the presence of serpin Z4. (a) The activity of 2 pmol of β‐amylase was assayed using the Betamyl substrate from Megazyme in the presence of different concentrations of serpin. The graphs are representative of three independent experiments. Graph (a) is presented as change in O.D. (b) As in (a) using the reducing sugar DNSA method. The enzymatic activity of β‐amylase in the presence of serpin beginning at the asterisk is significantly different from the no serpin control with a *p*‐value < .03. (c) Kinetic properties of β‐amylase with serpin Z4 and DTT. The maximal velocity (*V*_max_) and the substrate affinity (Km) are derived from the Lineweaver–Burk model. *V*_max_ is expressed in nmol maltose per minute, and Km is in mg/ml of starch. * The *V*_max_ of β‐amylase + serpin Z4 is significantly different from the Vmax of the β‐amylase control with a *p*‐value = .001. ** The *V*_max_ of β‐amylase+ Z4 + DTT 5 mM is significantly different from the Vmax of the β‐amylase + DTT 5 mM control with a *p*‐value = .001. The kinetic properties of β‐amylase were assayed in three independent experiments each one comprising three technical replicates

To investigate possible ramifications of the interaction between β‐amylase and serpin Z4, a dose–response activity curve for β‐amylase in the presence of increased serpin concentration was performed. Figure 4a shows the effect of serpin on β‐amylase enzymatic activity assayed with the Betamyl‐3 method. As the amount of serpin increases (abscissa axis), β‐amylase activity in the presence of excess substrate is significantly enhanced. The Betamyl‐3 method uses short‐chain substrate (p‐nitrophenyl‐β‐D‐maltotrioside) that requires auxiliary β‐glucosidase activity. Hence, we also examined the effect of serpin on β‐amylase using high MW starch substrate coupled to a direct assay of released reducing sugars. Figure 4b confirms the results using the DNSA technique. The concentration of serpin that was required for half of the maximal effect (EC50) is similar in both assays and was 1.8 · 10^−7 ^M and 3 · 10^−7 ^M for the Betamyl‐3 and DNSA methods, respectively. The EC50 value was obtained at a molar ratio of 1 β‐amylase to 1 serpin. The maximal increase in activity was obtained at a molar ratio of 1 β‐amylase to 10 serpins (Figure 4). Similar experiments carried out with bovine serum albumin (BSA) did not yield any change in β‐amylase activity indicating specificity (Fig. S5).

Notably, inspection of the dose–response of β‐amylase activation upon addition of serpin yields a curve that is consistent with the KD values obtained for the direct protein–protein interactions (Figure 3). To further dissect the effect of serpin on the kinetic characteristics of β‐amylase, Vmax and Km were estimated. As shown in Figure 4c, the addition of serpin at a 1:5 molar ratio led to a twofold increase in the Vmax independent of the presence of the reducing agent, DTT. Serpin Z4 had a slight negative effect on the affinity of β‐amylase to its substrate as indicated by the increase in Km.

**Figure 5:**
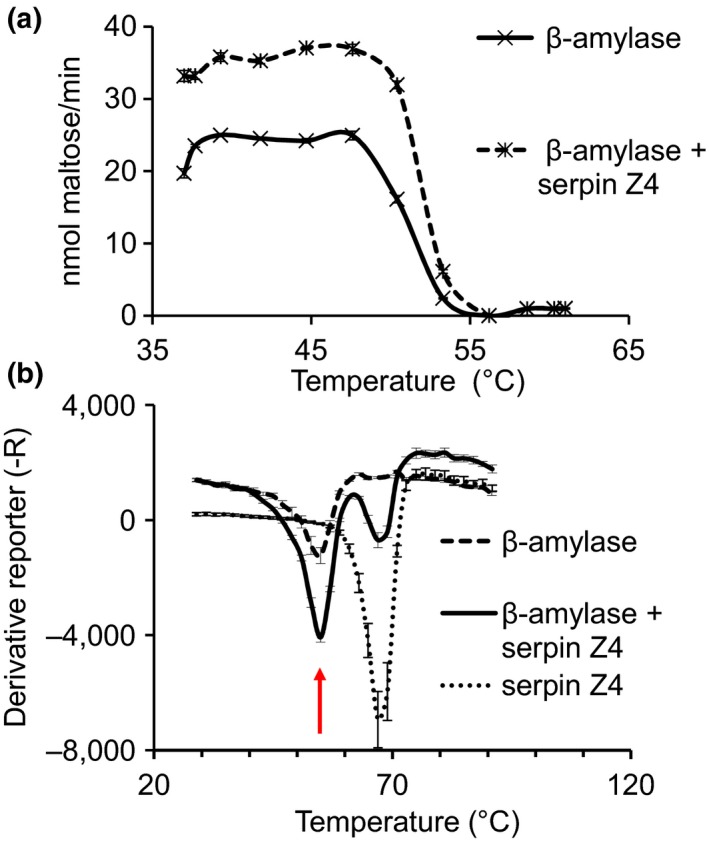
The effect of temperature on β‐amylase activity. (a) β‐amylase (2.5 pmol) was assayed by the DNSA method at different temperatures with or without serpin Z4 at a molar ratio of 1:5. The activity is presented in nmol of maltose released per minute of reaction. From 37°C to 53.3°C, the enzymatic activity of β‐amylase in the presence of serpin is significantly different from the control with a *p*‐value ≤ .03. The experiment was repeated three times with three technical replicates. (b) The thermal stability of β‐amylase with and without serpin Z4 was measured by thermal shift assay using sypro‐orange dye. The arrow indicates the melting temperature of β‐amylase. The graph is representative of three independent replicates

Temperature is a critical environmental parameter (e.g., during beer mashing) that affects the enzymatic activity of β‐amylase (Evans, van Wegen, Ma, & Eglinton, 2003). β‐amylase activity was assessed over a temperature gradient ranging from 37°C to 62°C, supplemented or not, with serpin Z4 (ratio of 1:5, β‐amylase: Z4; Figure 5a). Over a critical range of 37–48°C, the presence of serpin significantly improved β‐amylase activity. At 50°C, the activity of β‐amylase alone dropped by 40%, but with the addition of serpin Z4, the enzyme activity was enhanced by 60% compared with β‐amylase alone at optimal temperatures. Above 53°C, β‐amylase lost more than 80% of its activity with or without serpins. Thus, the presence of serpin elevates β‐amylase activity over a critical range of temperatures. However, the denaturing temperature for protein stability as determined by the “Thermal Shift” assay in Figure 5b showed that the intrinsic melting temperature of β‐amylase does not change in the presence of serpin Z4 and remains 55°C.

### 3.4 Impact of serpin on the redox‐related activity and polymerization state of β‐amylase

**Figure 6:**
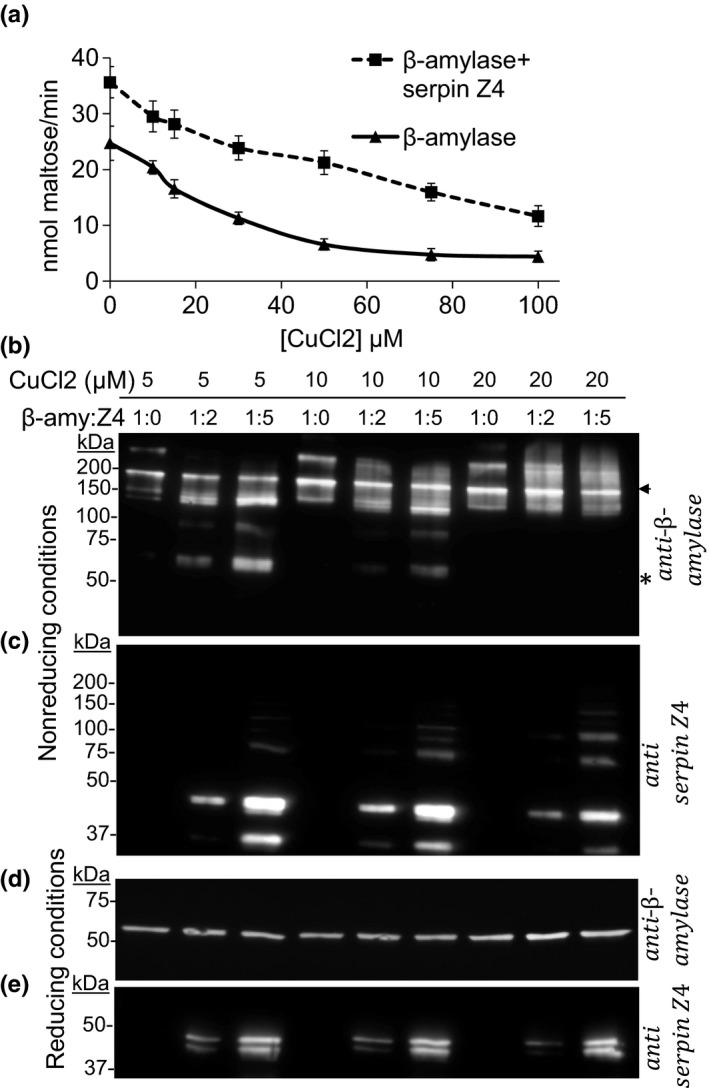
Enzymatic activity and immuno‐detection of recombinant β‐amylase and serpin under oxidizing conditions. (a) The activity of 2.5 pmol β‐amylase was assayed using the DNSA method with or without the addition of serpin Z4 at a 1:5 ratio upon increasing CuCl_2_ concentrations. (b–d, e) β‐amylase and serpin Z4 were mixed at the indicated ratios and treated with 5, 10, and 20 μM CuCl_2_ for 10 min. The proteins were fractionated in 10% SDS‐PAGE gel with (b, c) or without the addition of reducing agent, β‐mercaptoethanol (d, e) and blotted with antibody against β‐amylase (b, d) or against serpin Z4 (c, e). The asterisk indicates β‐amylase monomer size and the arrow polymeric forms. Western blots and enzymatic assays were replicated at least three times

Oxidative conditions were shown to play a role in β‐amylase activity (Seung et al., 2013; Sparla et al., 2006). It was therefore of interest to examine whether the presence of serpin impacts on the redox reactivity of β‐amylase. Oxidative conditions were simulated by the use of CuCl_2_ that oxidizes reactive cysteines inducing the formation of cysteine–cysteine bonds (Cavallini, De Marco, Duprè, & Rotilio, 1969) (Figure 6a). Without serpins, β‐amylase activity starts decreasing from 10 μM, until 50 μM at which point, less than 20% of the initial activity remains. The presence of serpins improves β‐amylase stability to oxidation, as after treatment with 50 μM CuCl_2_, β‐amylase was as active as the control β‐amylase without the oxidative agent. However, a gradual decrease in activity, although less steep, was observed due to CuCl_2_ even in the presence of serpin.

Grain development and germination seem to affect the polymerization state of β‐amylase (Figure 1), and these changes have been ascribed to shifts in the cellular redox milieu (Niku‐Paavola, Skakoun, Nummi, & Daussant, 1973). Therefore, it was of interest to examine the impact of serpins on the polymerization status of β‐amylase upon oxidation. To visualize this, recombinant proteins were combined at molar ratio of 1:0, 1:2, and 1:5 (β‐amylase:serpin Z4). The mixtures were then subjected to treatment with CuCl_2_. The proteins were fractionated on a SDS‐PAGE with or without reducing agent and developed by immunoblot with anti‐β‐amylase or antiserpin Z4 antibody (Figure 6b–e). Oxidative treatment caused β‐amylase and serpin Z4 to migrate in multiple higher molecular weight forms (Figure 6b and c). β‐amylase migrated in apparent dimer and trimer size at approximately 120 and 180 kDa, respectively; however, higher molecular weight migration forms also appeared. In the case of serpin Z4, dimeric (80 kDa) and higher order forms were visible, although to a lesser extent. Interestingly, the addition of serpins increased the amount of β‐amylase monomers, inhibiting the formation of high molecular weight β‐amylase. Notably, all polypeptides migrated at their monomeric size after the addition of the reducing agent, β‐mercaptoethanol (Figure 6d and e). Hence, β‐amylase and serpin Z4 polymerization are due to the formation of cysteine bonds in the oxidative environment. The results show that the multiple polypeptide states of β‐amylase are dictated by the oxidation level and that increasing levels of serpin Z4 reduce the formation of higher molecular weight β‐amylase.

### 3.5 β‐amylase mutants exhibit a differential response to oxidation

To investigate the participation of specific cysteines residues in β‐amylase contributing to its polymerization, two modified β‐amylases were generated. The β‐amylase WT used in this study originates from cv. Harrington and contains a cysteine at position 115 (Chiapparino, Donini, Reeves, Tuberosa, & O'Sullivan, 2006). It was modified by a C115R replacement. The second modification was the removal of the C‐terminus of β‐amylase from the glycine at position 489 (Trunc β‐amylase). This removes a cysteine at position C503. These cysteines were chosen as they are known to impact on the thermostability and enzymatic properties of β‐amylase (Ma, Eglinton, Evans, Logue, & Langridge, 2000; Ma et al., 2001). Moreover, C503 was found to be the target of thioredoxins released in the endosperm upon germination (Hagglund et al., 2010).

**Figure 7:**
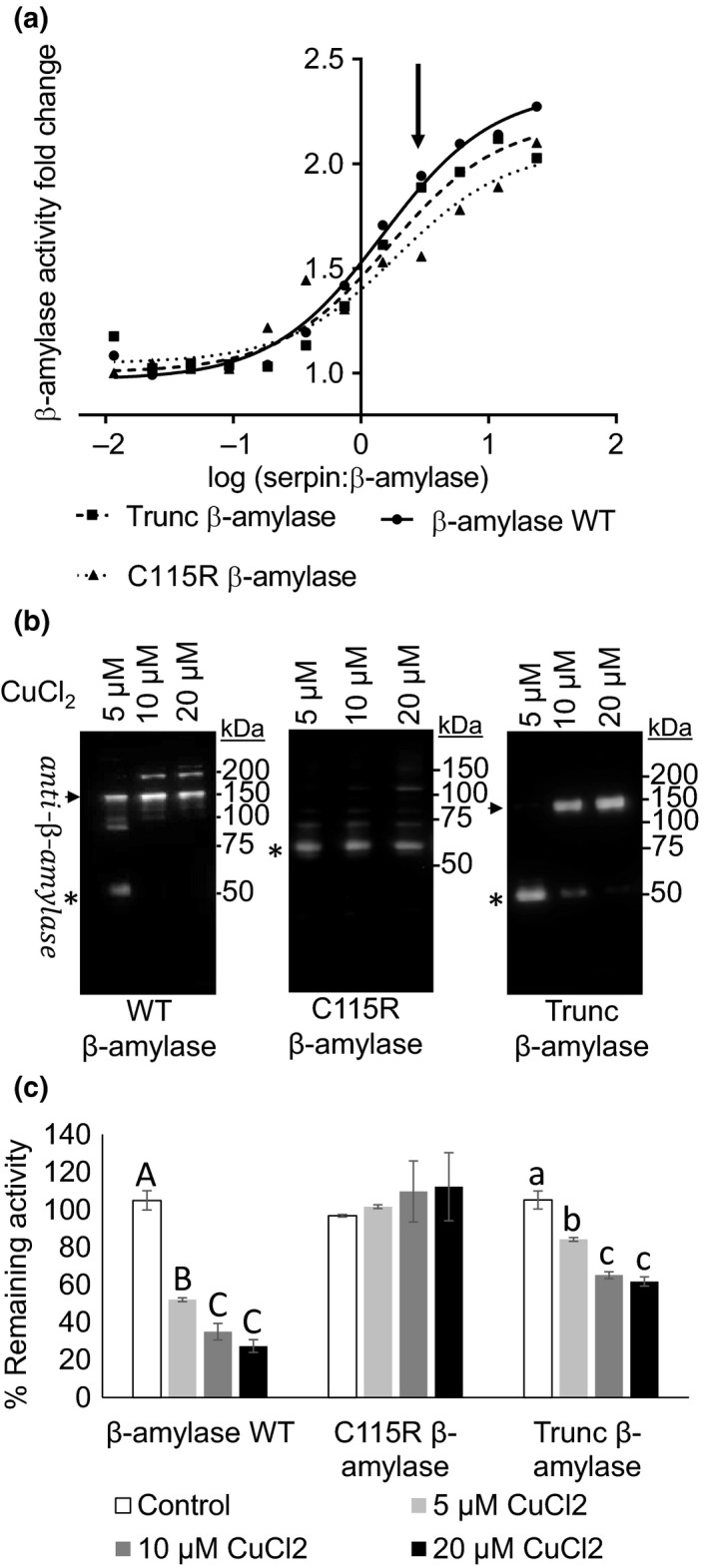
Enzymatic activity and immuno‐detection of wild type and mutant forms of β‐amylase in the presence of serpin Z4. (a) Activity of 2 pmol of β‐amylase with increasing concentrations of serpin Z4 assayed by the DNSA method. Assays were on WT, C‐terminus truncated β‐amylase (Trunc β‐amylase) and the C115R amino acid replacement. The X axis represents the ratio of serpin Z4 to the β‐amylases. The Y axis expressed the fold change in the β‐amylase activity with serpin over the activity of the β‐amylase without serpin. Serpin Z4: β‐amylase ratios range between 20:1 and 0.04:1. The arrow indicates a ratio of 1:5. (b) Immunoblot analysis of recombinant β‐amylase wild type and mutants as in a. Proteins (0.6 μg) were treated with 5, 10, and 20 μM of CuCl_2_, for 10 min and fractionated on a 10% SDS nonreducing PAGE gel and immunobloted with β‐amylase antibody. (c) The residual activity of β‐amylase compared to no treatment after addition of CuCl_2_ as in b assessed by the DNSA method. Capital letters indicate significant differences in β‐amylase WT‐treated enzyme, lower cases letters, significant differences in Trunc β‐amylase‐treated enzyme, no statistical differences were found in the C115R β‐amylase‐treated enzyme. The results were analyzed by performing Tukey‐Kramer HSD tests. The asterisk indicates the monomeric forms of the three β‐amylases and the arrow, the polymers of β‐amylase WT and Trunc β‐amylase. Western blots and enzymatic assays were replicated at least three times

The effect of serpin Z4 was evaluated on β‐amylase activity of WT, C115R, and Trunc β‐amylase (Figure 7a). As shown in Figure 7a, WT and all the variants displayed similar activity enhancement with no significant statistical difference as result of the presence of serpin Z4. For example, at a 5:1 ratio (arrow; Figure 7a), the addition of serpin Z4 improves all β‐amylase to the same degree. Hence, the action of serpin on β‐amylase activity was unaffected by modifications of these cysteines.

To investigate the ramifications of the mutations on the polymerization patterns, 5–20 μM of CuCl_2_ was applied to the β‐amylase variants. Without CuCl_2_, each polypeptide migrated at the expected molecular weight size of its monomeric form (Fig. S6). However, as observed in Figure 7b, while β‐amylase WT rapidly polymerized due to increasing CuCl_2_, the C115R variant remains mainly in its monomeric form even at the highest concentration of CuCl_2_ (20 μM). Although WT β‐amylase appears smaller than expected, LC/MS/MS analysis was conducted on the protein and showed the expected size value (Fig. S7). The Trunc β‐amylase variant also showed polymerization patterns that were distinct from the WT. Beside the monomeric form, one additional band is spotted on the Western blot as a result of oxidation. However, its polymeric migration size as determined by nondenaturing gel fractionation is 140 kDa, a value that is larger than the predicted dimer size (117 kDa) but smaller than the expected trimer size (176 kDa). In addition, the Trunc β‐amylase variant appeared to be less susceptible to oxidation than the WT as it remained as a monomer at 5 μM CuCl_2_. When the percent of residual amylase activity in the presence of CuCl_2_ was measured, both WT and Trunc β‐amylase showed a decrease in activity but not the C115R variant (Figure 7c). Thus, the C115R β‐amylase, that is less inclined to form polymers, shows less sensitivity to oxidation indicating that the decrease in enzymatic activity might be a consequence of the polymerization process. Although the three forms of β‐amylase exhibited distinct and differential responses to CuCl_2_, they all responded to serpin as the WT, with enhanced enzymatic activity as measured in Figure 7a. Hence, serpin can improve β‐amylase enzymatic activity independent of its ability to modify its oligomerization state.

## 4 DISCUSSION

Serpin Z4 and β‐amylase are highly abundant seed proteins in many barley cultivars (Finnie et al., 2004). Similar to storage proteins, their content increases in the endosperm of developing grains (Sreenivasulu et al., 2006) and accumulate as matrix‐bound proteins. Here, we show that both proteins display comparable accumulation profiles during seed maturation and germination and colocalize to the cytosol in a heterologous expression system (Figures 1 and 2). As the grain ripens and cell death proceeds in the endosperm (Young & Gallie, 2000), cytosolic proteins agglutinate to coat the amyloplast‐localized starch granules (Borén et al., 2004; Wang et al., 2011). Consistent with our results, this coating would include serpin Z4 and β‐amylase. During the later stages of grain filling and the early stages of germination, both β‐amylase and serpin Z4 are detected mostly as a bound nonfree fraction or as high molecular weight protein in the free fraction (Figure 1). As they accumulate, the polymerization profiles of these two barley proteins are consistent with the creation of disulfides bridges to form homopolymers as has been reported for proteins in ripening wheat grains (De Gara, de Pinto, Moliterni, & D'Egidio, 2003). Significantly, serpin Z4 and β‐amylase were detected among the multiple putative targets of thioredoxins, that is, reducing agents released from the aleurone layer during germination (Hagglund et al., 2010). Thus, serpin Z4 and β‐amylase share common spatio‐temporal localization characteristics that would promote interaction during grain development and germination.

Using different techniques, serpin Z4 was shown here to interact with β‐amylase with a binding constant (KD) of approximately 10^−7^ M (Figure 3). This interaction is in the range measured for a variety of chaperones (Schweiger, Soll, Jung, Heermann, & Schwenkert, 2013). Importantly, in the presence of serpin Z4, the enzymatic activity of β‐amylase is improved significantly. The improvement was specific as the addition of BSA had no effect (Fig. S3). Thus, β‐amylase activity exhibits a sigmoidal curve of activity upon the addition of serpin (Figure 4); a curve that typifies agonistic interactions between proteins (DeLean, Munson, & Rodbard, 1978). A ratio of 1 β‐amylase to 5 serpins increased the maximal velocity by twofold and protected β‐amylase activity from heat or oxidation stress (Figures 5 and 6). It was previously reported that a serpin Z4‐deficient barley line showed more diastatic power, that is, was more efficient in converting starch (Iimure, Kimura, et al., 2012). However, as diastatic power is a gross measurement taking into consideration multiple enzymatic activities including β‐amylase, α‐amylases and limit dextrinase activity, it may be that, in that case, other qualities compensated for the reduced level of serpin.

Under oxidizing conditions, β‐amylase WT rapidly polymerized and lost significant activity (Figure 7). The kinetics of polymerization shows that while the C115R mutation stabilized β‐amylase in its monomeric form, the Trunc β‐amylase mutant still polymerized but in a manner distinct from the WT. A number of β‐amylase variants exist in barley. The SD1 and SD2 β‐amylase isotypes are commonly found in malting‐type barley (Allison & Swanston, 1974; Evans, Wallace, et al., 1997). The substitution of cysteine at position 115 by an arginine results in the conversion of SD1 to the SD2 type (Ma et al., 2002). SD1 is more thermostable than SD2 but has lower affinity to its substrate (Ma et al., 2001). Moreover, a SD1 type β‐amylase from cv. Pirkka was shown to polymerize spontaneously (Niku‐Paavola et al., 1973) in a manner similar to the WT described in this work (also a SD1 type). The C115R mutation transforms the SD1 β‐amylase into a SD2 type and as shown here prevents the formation of high molecular weight moieties (Figure 7) identifying a cysteine critical for higher order polymerization. In this scenario, C115 could serve as a hub for cysteine bonds formation. Engineered removal of the C‐terminus eliminates C503 yielding a more stable β‐amylase (Figure 7). This is consistent with the finding that reduction of the C503 by thioredoxins (Hagglund et al., 2010) or its removal by proteases (Guerin et al., 1992; Lundgard & Svensson, 1987) efficiently solubilizes the bound β‐amylase. The resulting processed β‐amylase was found to be more thermostable but otherwise retained the same kinetic parameters (Ma, Eglinton, et al., 2000). The decrease in β‐amylase activity due to oxidation reported here differs from that reported for the redox‐sensitive Arabidopsis, trBAMY. The Arabidopsis trBAMY, a thioredoxin regulated β‐amylase, does not form high molecular weight complexes, suggesting that in that case intramolecular disulfide bonds account for the control of enzymatic activity (Sparla et al., 2006).

Under oxidizing conditions, serpin Z4 interaction impacts on the enzymatic activity of WT β‐amylase in two ways. Firstly, the interaction between β‐amylase and serpin Z4 represses the formation of multimeric β‐amylase (Figure 6b and c). Presumably such polymers are less active (Figure 7c). Secondly, its intrinsic ability to activate β‐amylase is evidenced in the mutant C115 that lacks the ability to polymerize but is nevertheless enhanced by serpin. How serpin and β‐amylase interactions interfere with polymerization is not known. Based on β‐amylase structure (PDB ID: 2XFR), C115 is located on the outer surface far from the active site. One can speculate that in the topography of its interaction, serpin Z4 specifically interferes with the ability of C115 to form cysteine bonds perhaps by physical contact in that area.

The function for serpin Z4 in grains of barley as a chaperone‐like stabilizer of β‐amylase that enhances enzyme activity and favors monomeric forms differs from classical chaperones that operate in protein folding pathways. Yet, in mammals, the serpin family was found to participate in a wide range of activity beside protease inhibition (Gettins, 2002). For example, Hsp47 is a serpin whose function is to prevent the aggregation of protocollagen, a collagen precursor particularly abundant in the ER (Ito & Nagata, 2017; Natsume, Koide, S‐i, Hirayoshi, & Nagata, 1994). Hsp47 stabilizes their cognate protein partners by binding to the triple helix of type I collagen to prevent further aggregation (Koide et al., 2006; Widmer et al., 2012).

Despite the importance of starch breakdown for providing a source of energy, β‐amylase and serpin Z4 are not absolutely required for germination of barley seeds, and barley cultivars with variable amounts of both proteins can be found in nature (Iimure, Kimura, et al., 2012; Kihara, Kaneko, Ito, Aida, & Takeda, 1999). However, barley strains that are dedicated to the production of beer require functional α‐amylase and β‐amylase to be present in the mashing step for the release of maltose units favored by fermenting yeast. Serpin Z4 was also found to contribute to beer quality by improving foam stability along with other beer proteins such as LTPs (Evans & Hejgaard, 1999). Hence, selection pressure by thousands of years of human use has likely contributed to shaping the attributes of today's barley varieties.

This work shows that serpin Z4 interacts with β‐amylase and optimizes its activity. In the presence of serpin Z4, the maximal catalytic velocity of β‐amylase is doubled, and β‐amylase was found to stabilized by serpin Z4 during heat and oxidative treatments. Thus, serpin Z4 shows novel noninhibitory attributes by displaying chaperone‐like activity toward β‐amylase.

## AUTHOR CONTRIBUTION

M.C. and R.F. conceived the experiments. M.C. carried them out. M.C. and R.F. wrote the manuscript.
