# Supplementary material for: Noncanonical interactions between serpin and β‐amylase in barley grain improve β‐amylase activity in vitro
Source: Plant Direct. 2018 May 8;2(5):e00054. doi: 10.1002/pld3.54 (PMC6508567; doi:10.1002/pld3.54)
Supplement: Supplementary file 1 [file PLD3-2-e00054-s001.pdf]

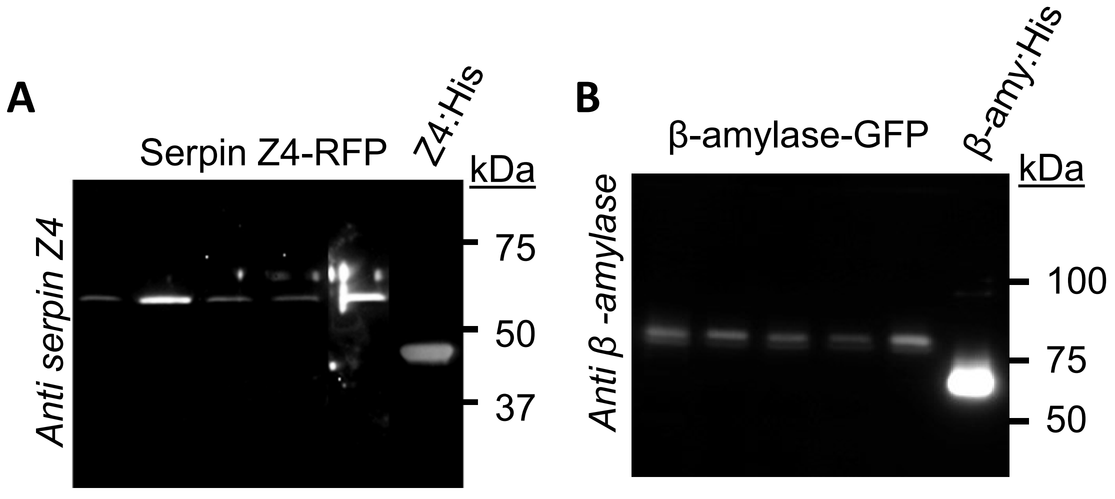

Figure S1. *Nicotiana Benthaniama* leaves transiently expressing serpin Z4-RFP and β-amylase-GFP. Proteins were extracted from the leaves and immuno-blotted with serpin Z4 and β-amylase antibody. Independent experiments (5) were analyzed. In the right-most lane, recombinant serpin Z4 and β-amylase were fractionated (Z4:His; β-amy:His), respectively. A, The expected sizes of serpin Z4 and serpin Z4-RFP are 45 kDa and 69 kDa, respectively. B, The expected sizes of β-amylase and β-amylase-GFP are 60 kDa and 85 kDa, respectively.

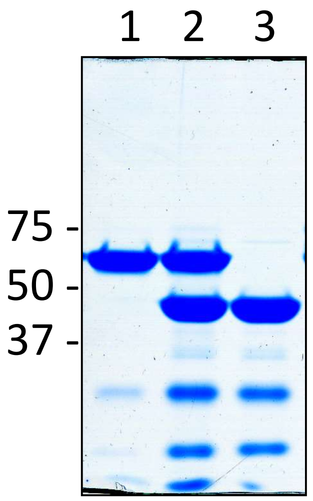

Figure S2. 10% SDS Page gel of 10 µg of β-amylase (1), 10 µg of both β-amylase and serpin Z4 (2) and 10 µg of serpin Z4 (3) in presence of 15% β-mercaptoethanol.

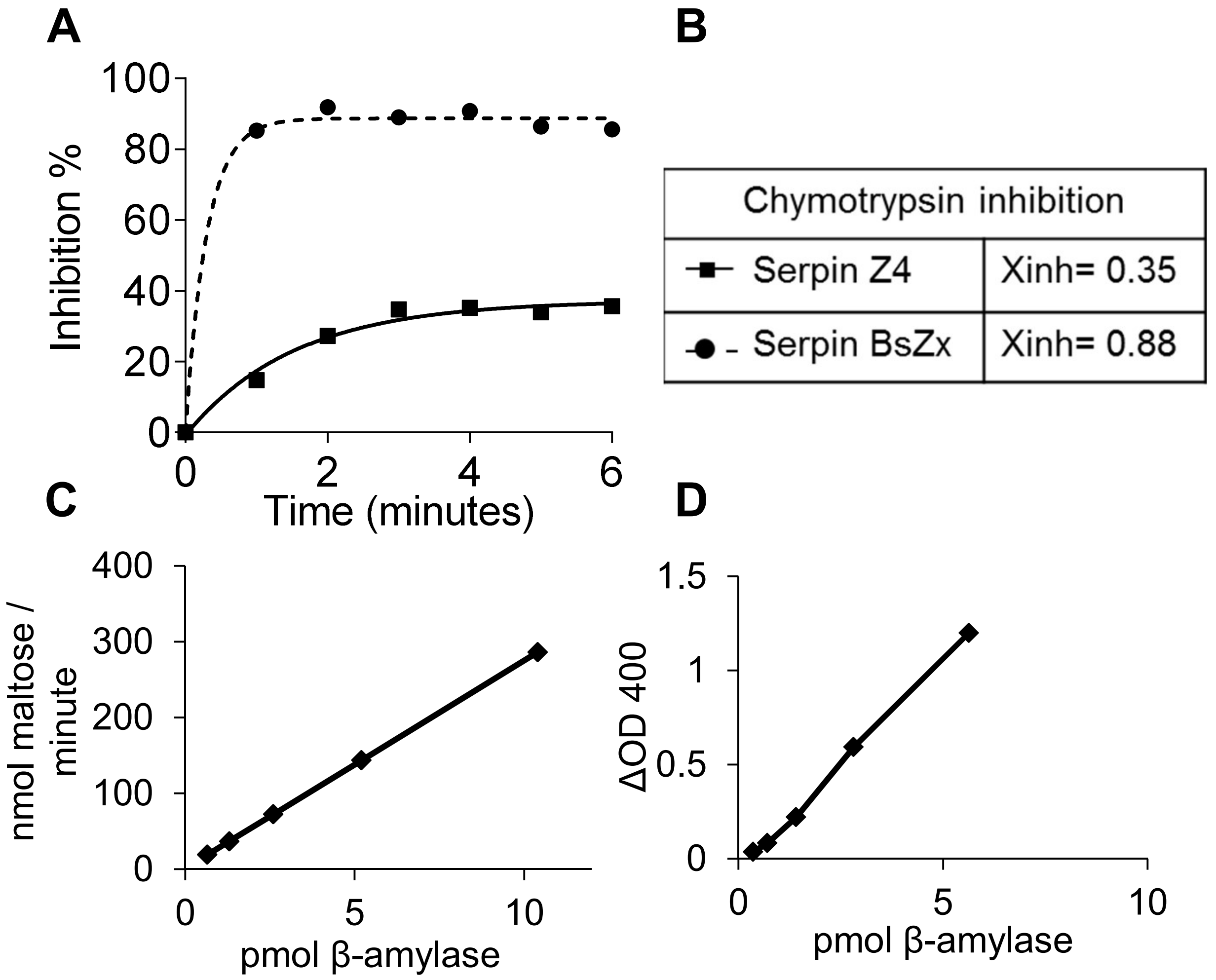

Figure S3. The recombinant proteins, serpin Z4 and β-amylase (HV bamy1) from *Hordeum vulgar* cv. Harrington were tested for their intrinsic activities. A, Recombinant serpin Z4 was tested for its ability to inhibit chymotrypsin. As positive control, the inhibitory activity of serpin BsZx from barley was also measured. BsZx is known to inhibit chymotrypsin at a Xinh of 0.9. (Dahl, Rasmussen et al. 1996). B, the Xinh (factor of inhibition) represents the fraction of serpin in complex with the protease at equilibrium. C and D, The enzymatic activity of the recombinant β-amylase was measured by two independent methods, the reducing sugar method (DNSA) in C and the Betamyl-3 method in D.

A.

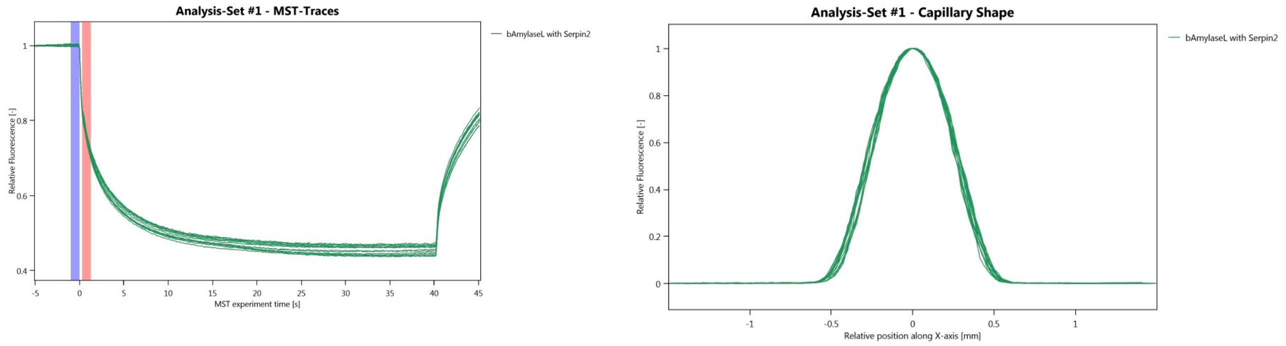

B.

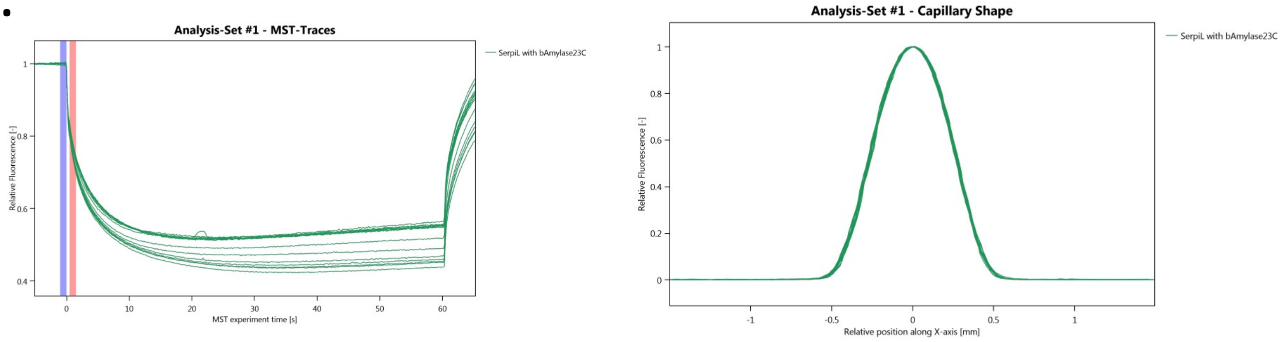

Figure S4. Exemplary MST traces and capillary shapes. A, β-amylase is attached to the fluorescent probe and B, serpin is attached to the fluorescent probe. The graphs are representative of 3 independent replicates in each reciprocal labeling.

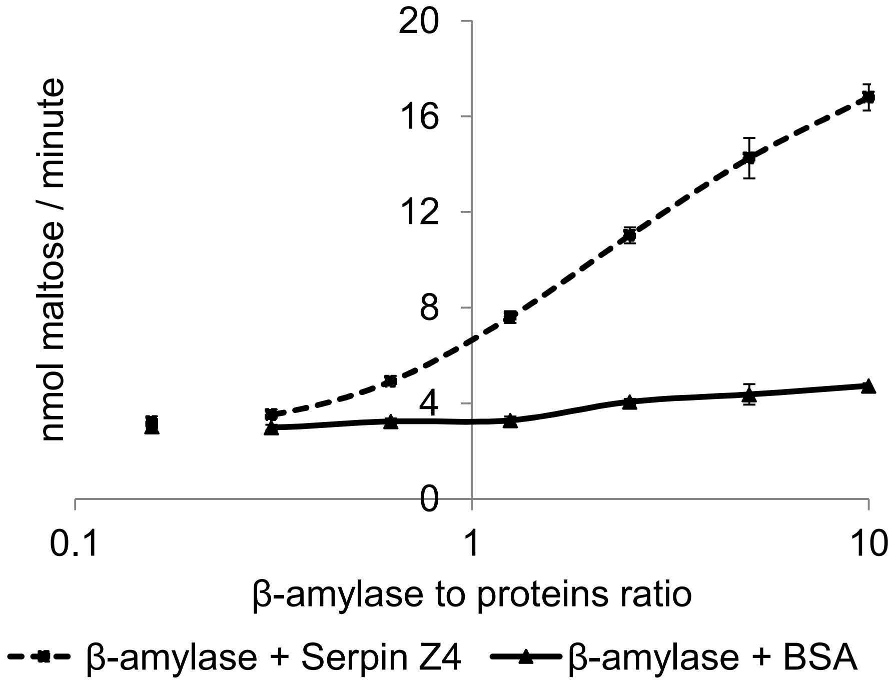

Figure S5. The effect of serpin Z4 and BSA on β-amylase activity. The enzymatic activity of 2 pmol of β-amylase treated with 50 µM of $CuCl_2$ was measured with serpin Z4 and with BSA as control.

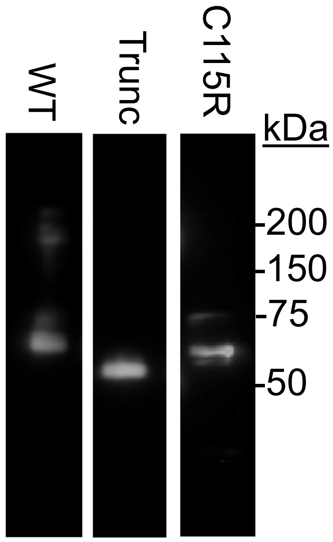

Figure S6. Western Blot of WT β-amylase , Truncated β-amylase and C115R β-amylase in PBS fractionated under non-reducing conditions.

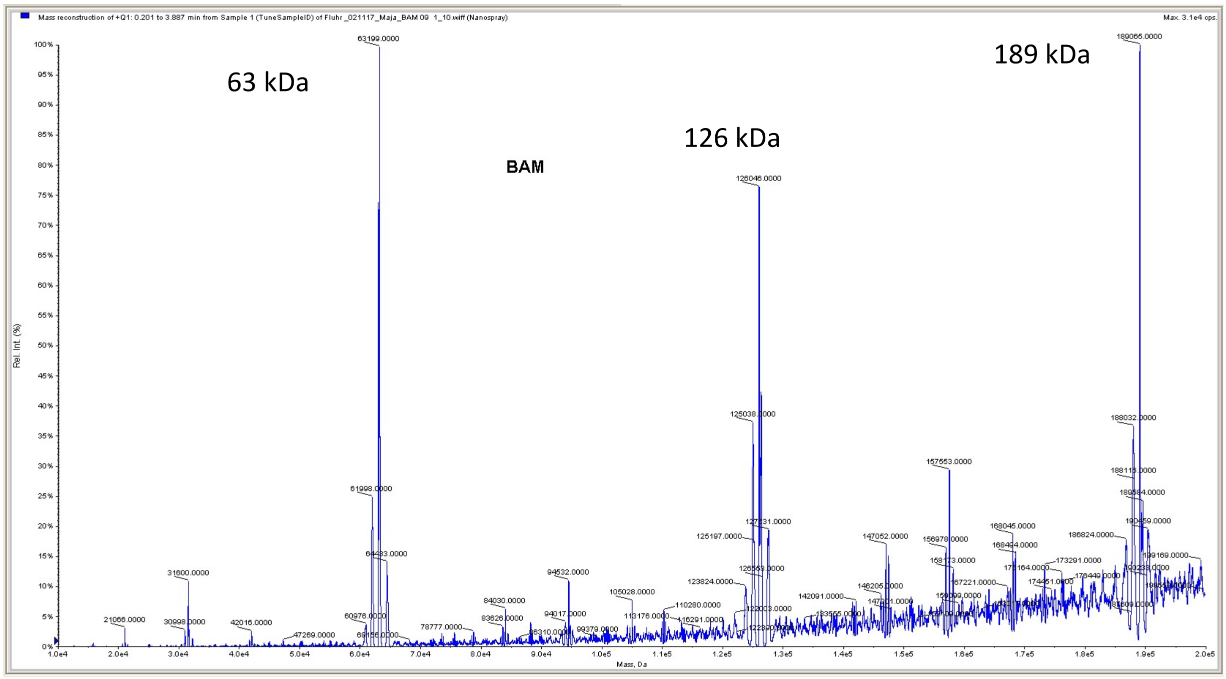

Figure S7. Q-trap LC/MS/MS of β-amylase WT. The 3 peaks correspond to the monomeric form (63kDa), the dimeric form (126kDa) and the trimeric form (189kDa)

		Forwar primers	Reverse primers
**Serpin Z4**	Isolation from Cdna	ATGGCAACCACCCTCGCCAC	TTATGCAGATATGAGGGGATTGGTCA
	Cloning in pet 28A, BamHI XhoI	ACTGTTACGGATCCGCAACCACCCTCGCCA	ATCGGCCTCGAGTTATGCAGATATGAGG
	RFP:Z4 in pART7		
	RFP (BamHI)	ATCGTCTCGAGATGTCCTCCGAGGACG	GATCCTGGATCCTGCGGCGCCGGTGGAG
	Z4 (BamHI)	GATAGGATCCTCCGCAACCACCCTCG	TCCGGCCCGGGTTATGCAGATATGAGG
	Cloning RFP:Z4 in pART7, XhoI, XmaI	ATCGTCTCGAGATGTCCTCCGAGGACG	TCCGGCCCGGGTTATGCAGATATGAGG
**β-amylase 1**	Isolation from Cdna	ATGGAGGTGAACGTGAAAGGCAACTATG	TTACATGGTGGCAGGGAGCTCCCCACC
	Cloning in pet 28 BamHI , XhoI	ATAGGATCCATGGAGGTGAACGTGAAAGGC	ATTACTCGAGTTACATGGTGGCAGGGAG
	β-amylase: GFP in pART7		
	β-amylase Overlaping PCR	AGAGGATCCGAGGTGAACGTGAAAG	CTCGCCCTTGCTCACCATGGTGGCAGGGAGC
	GFP Overlaping PCR	CTCCCTGCCACCATGGTGAGCAAGGGCGA	AATCATCTAGACTTGTACAGCTCGTCC
	Cloning β-amylase: GFP in pART7,BanHI, XbaI	AGAGGATCCGAGGTGAACGTGAAAG	AATCATCTAGACTTGTACAGCTCGTCC
**β-amylase mutants**	C115R β-amylase by overlaping PCR	GGGACGTCGGCACGCGTGATCCCGACATTTTC	GAAAATGTCGGGATCACGCGTGCCGACGTCCC
	Trunc β-amylase BamHI XhoI	ATAGGATCCATGGAGGTGAACGTGAAAGGC	ATTACTCGAGTTAAGTAGGGCCTACTGGCA

Table S1. List of primers
