# Supplementary material for: Noncanonical interactions between serpin and β‐amylase in barley grain improve β‐amylase activity in vitro
Source: Plant Direct. 2018 May 8;2(5):e00054. doi: 10.1002/pld3.54 (PMC6508567; doi:10.1002/pld3.54)
Supplement: Supplementary file 2 [file PLD3-2-e00054-s002.pdf]

March 23 2108

Dear Editor,

We have provided answers (below) and where necessary corrections (in the text and Figures) to all the reviewers comments.

Thank you for your consideration and effort.

Sincerely, Authors.

<u>Editor comments</u>

Title Change:

<u>Line 13:</u> Title: Non-canonical interactions between serpin and β-amylase in barley grain improves β-amylase activity *in vitro*

comments of the referees and add a significance statement/ conclusion in the discussion:

The responses to the comments appear below. A summary sentence was added in the Discussion:

<u>Line 460:</u> Serpin Z4 interacts with β-amylase and optimizes its activity. In the presence of serpin Z4, the maximal catalytic velocity of β-amylase is doubled and β-amylase was found to stabilized by SerpinZ4 during heat and oxidative treatments. Thus, serpinZ4 shows novel non-inhibitory attributes by displaying chaperone-like characteristics towards β-amylase.

<u>Reviewer 1</u>

1) *Figures 1, 6 and 7; description (legend) for the multiple bands seen on the blots would be helpful (which one corresponds to the monomer, aggregate, truncated protein,....).*

<u>Changes included in all relevant figures and their legends.</u>

2) *Figure legends; could the authors provide replication info for all the figures? Is present for most but not all presented data.*

<u>Information that all experiments are representative of at least 3 repeats is included in all Figure legends where relevant.</u>

3) *Figure 3, can the authors provide exemplary MST traces?*

Now provided:

Line 225: Typical MST traces and capillary shapes are shown in Supp. Fig. S4.

Line 597: Supp. Figure S4. Exemplary MST traces and capillary shapes.

4) *Figure 7A. However all three beta-amylase variants are clearly induced by serpin the FC is lower for the C115R and truncated beta-amylase. Can the authors comment on that?*

While the appearance may be that the FC is lower for C115, it is not statistically significant. That specific statement is now added to the text, line 341.

Reviewer 3

1) *Page 5 line 140: change (1-4) α bond in α-1,4-glycosidic bond*

Changed in Line 140

2) *All along the manuscript: check the spelling Kd, KD, Ka, Vmax etc.*

Note that throughout we have changed Kd to KD (the disassociation constant) and Kdiss refers only to measurement BIACORE rate of disassociation.

3) *Because must of the work have been performed on purified recombinant Serpin Z4 and β-amylase, include the gel images of the different western blots in supplementary material is strongly suggested.*

The amounts used for the western blots would not be detected by stain, hence stained gels are not included. However, we now include one stained gel in Supp. Fig. S2 to show the purified recombinant proteins.

Line 203: The gel image of the proteins is shown in Supp. Fig. S2.

4) *Page 16, line 468: add all the primer sequences that have been used*

Supp. Table S1. List of primers has been added.

5) *Page 16, line 474: add the setting of the confocal microscope*

Added in Line 508:

6) *Page 16, line 477: see comment 4*

Added, see comment 4.

7) *Fig 7, Panel B, 1st blot: the band corresponding to β-amylase is smaller than expected (50 kDa instead of 60kDa). Give an explanation*

<u>Line 350:</u> Although WT β-amylase appears smaller than expected by gel migration, LC/MS/MS analysis was conducted on the protein and it showed the expected size value (Supp. Fig. S7).
